# Selective Oxidation of Simple Aromatics Catalyzed by Nano-Biomimetic Metal Oxide Catalysts: A Mini Review

**DOI:** 10.3389/fchem.2020.589178

**Published:** 2020-10-26

**Authors:** Wondemagegn H. Wanna, Damodar Janmanchi, Natarajan Thiyagarajan, Ravirala Ramu, Yi-Fang Tsai, Steve S. F. Yu

**Affiliations:** ^1^Institute of Chemistry, Academia Sinica, Taipei, Taiwan; ^2^Sree Dattha Institute of Engineering and Science, Hyderabad, India

**Keywords:** benzene, phenol, *p*-benzoquinone, hydrogen peroxide, toluene, metal oxide, nanocatalysts

## Abstract

The process of selective oxy-functionalization of hydrocarbons using peroxide, O_3_, H_2_O_2_, O_2_, and transition metals can be carried out by the reactive oxygen species such as hydroxyl/hydroperoxyl radical and/or metal oxygenated species generated in the catalytic reaction. Thus, a variety of mechanisms have been proposed for the selective catalytic oxidation of various hydrocarbons including light alkanes, olefins, and simple aromatics by the biological metalloproteins and their biomimetics either in their homogeneous or heterogeneous platforms. Most studies involving these metalloproteins are Fe or Cu monooxygenases. The pathways carried out by these metalloenzymes in the oxidation of C–H bonds invoke either radical reaction mechanisms including Fenton's chemistry and hydrogen atom transfer followed by radical rebound reaction mechanism or electrophilic oxygenation/O-atom transfer by metal-oxygen species. In this review, we discuss the metal oxide nano-catalysts obtained from metal salts/molecular precursors (M = Cu, Fe, and V) that can easily form *in situ* through the oxidation of substrates using H_2_O_2(aq)_ in CH_3_CN, and be facilely separated from the reaction mixtures as well as recycled for several times with comparable catalytic efficiency for the highly selective conversion from hydrocarbons including aromatics to oxygenates. The mechanistic insights revealed from the oxy-functionalization of simple aromatics mediated by the novel biomimetic metal oxide materials can pave the way toward developing facile, cost-effective, and highly efficient nano-catalysts for the selective partial oxidation of simple aromatics.

## Introduction

The technology of “advanced oxidation processes” essentially applies Fenton's chemistry for wastewater treatment (Andreozzi et al., 1999; Pignatello et al., 2006). The primary oxidants including O_3_, H_2_O_2_, O_2_, transition metal oxo-species, light, and electrical energy are employed to remove the organic (and sometimes inorganic) wastes in wastewater *via* the reactive oxygen species (ROS) and/or UV light (Deng and Zhao, 2015). These remediation processes can also be properly controlled and applied for the selective catalytic oxidation of hydrocarbons by designing variable homo- and heterogeneous catalyst systems to achieve green and environmentally benign fine chemical production (Ottenbacher et al., 2020; Suib et al., 2020).

In addition to accumulate ROS, it has been believed that the transition metals such as M = Fe, Cu, Co, and Mn in H_2_O_2(aq)_ can induce the metal oxygenates for the hydrocarbon activation (Sawyer et al., 1996). In fact, Fenton's chemistry can both possibly occur with the Haber–Weiss reaction mechanism (Equation 1) (Walling, 1975; Pignatello et al., 2006) for the accumulation of HO·/HOO· and for the formation ferryl-oxo species *via* Bray–Gorin mechanisms (Equation 2) (Bossmann et al., 1998; Enami et al., 2014). Interestingly, recent reports indicated the observation of Fenton's chemistry at the aqueous interface by mass spectrometry where there was no HO· observed but several ferryl-oxo species including dimeric forms were detected (Enami et al., 2014). The dimeric ferryl-oxo species are actually more reactive than the monomeric ferryl-oxo species for O-atom transfer (OAT) reaction. These further progresses in Fenton's chemistry have provided a significant insight to indicate the importance of the electronic coupling interplaying among the iron oxide clustering sites (Enami et al., 2014).


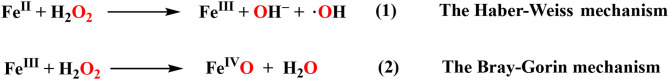


**Figure 1:**
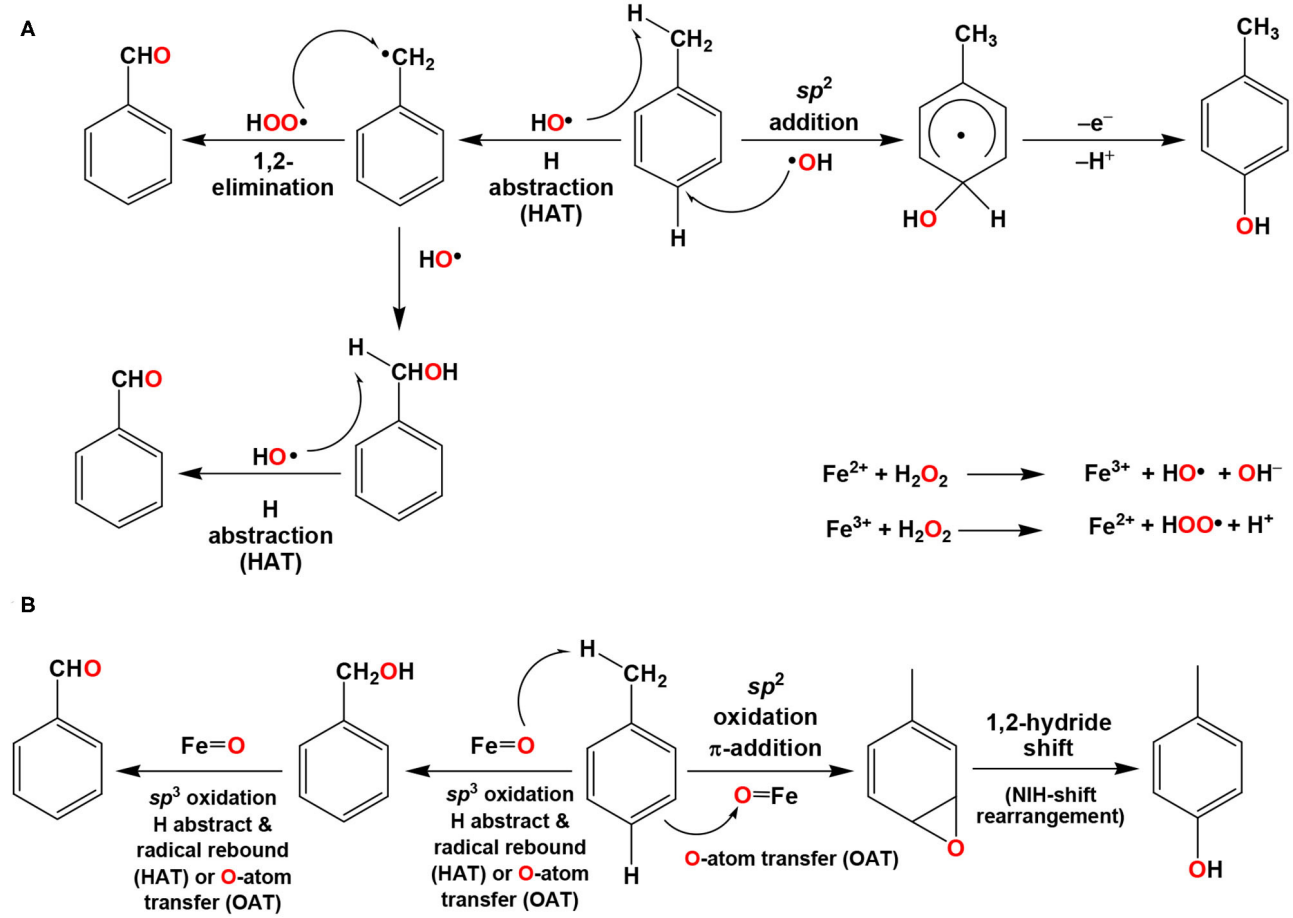
Ring or chain activation of toluene by **(A)** ROS accumulated by Fenton's reactions or **(B)** high valent metal oxygenate species in metalloproteins. The mechanism **(B)** can also be proceeded by the Bray–Gorin mechanism. Reproduced from Ramu et al. (2017). Copyright 2017 Elsevier B.V.

For the selective oxidation of *sp*^2^- *vs. sp*^3^-carbons, “toluene” can be considered as a great example to illustrate the reaction behaviors mediated either by ROS or transition metal oxygenated species. If the reactions are mediated by ROS of HO· or HOO·, it may proceed either by the hydrogen atom transfer (HAT) of C–H bond in the methyl group and then radical rebound to form benzyl alcohol/benzyl hydroperoxide or direct addition of HO· to the *sp*^2^ carbon center of *o*- or *p*-positions of the methyl substituent (Figure 1).

On the other hand, it has been long considered that the aromatic ring (*sp*^2^ carbon) oxygenation mediated by the metalloproteins such as cytochrome P450 and toluene monooxygenase (ToMO) are proceeded with the formation of arene oxide and/or σ-complex intermediates *via* OAT then 1,2-hydride shift (NIH shift rearrangement) toward the formation of phenolate products (Guroff et al., 1967; Boyd et al., 1972; Jerina and Daly, 1974; Bassan et al., 2003; de Visser and Shaik, 2003; Mitchell et al., 2003). With respect to *sp*^3^ C–H bond activation, the oxidation may directly be conducted by the high valent iron oxo or ferryl-oxo species either through HAT or OAT reaction mechanism (Bassan et al., 2003; de Visser and Shaik, 2003). In any case, the formation of benzaldehyde can be subsequently produced by oxidation through HAT and radical rebound or OAT toward the methylene C–H bond of benzyl alcohol (Figure 1). The bond dissociation energy (BDE = 86–89 kcal/mol) of C–H bond in methyl substituent of toluene is much lower than the ring activation of toluene (BDE = 109–112 kcal/mol) (Bassan et al., 2003; de Visser and Shaik, 2003).

**Figure 2:**
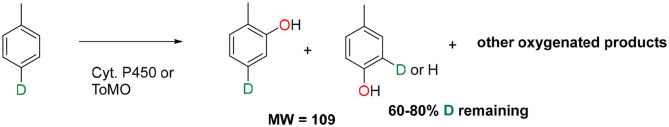
The *para*-hydrogen/deuterium in [4-^2^H_0, 1_]toluene can be activated by metalloprotein *via* 1,2-hydride shift after OAT to have significant deuterium remaining in the *p*-cresol product.

The significant mechanistic feature for OAT ring oxidation can be easily determined by enriched aromatics with a deuterium atom at the site/position for oxidation (Ramu et al., 2017). For toluene oxidation using H_2_O_2(aq)_, the *sp*^2^ activated site usually appeared either at the *o*- or *p*-position; therefore, [4-^2^H_0, 1_] toluene can be synthesized from 4-bromo-toluene in D_2_O (Morimoto et al., 2015) and used as a mechanistic probe to evidence the OAT mechanism if there is significant proportion of deuterium that remained in the molecules (Figure 2). Usually, the NIH shift ratios for metalloproteins can be up to 60–80% (Guroff et al., 1967; Boyd et al., 1972; Siegmund and Kaufman, 1991; Mitchell et al., 2003).

It is worth noting that in the past study, the addition of CH_3_CN to Fenton's reaction mixtures can enhance the NIH shift ratios from <5%, for Fe^II^ in aqueous solution, *i.e*., Fenton's oxidation, up to 50% (Castle et al., 1980; Kurata et al., 1988). These phenomena have made us wonder whether the corresponding reaction conditions can significantly accumulate any metal oxygenated species like metalloproteins of cytochrome P450 or ToMO. If so, then, how can we control the accumulated reactive oxygenated species for selective *sp*^3^ C–H *vs. sp*^2^ C–H bond oxidation *via* HAT or π-bond activation *via* OAT?

## Selective Oxidation of *sp*^2^ and *sp*^3^ C–H Bond Oxidation of Toluene by Nano-Iron Oxide Particles

Molecular iron complexes including polycyclic cage-like compounds, biomimetic model complexes, and encapsulated within zeolite cages have been shown with high catalytic activity in the oxidation of hydrocarbons including alkanes and aromatics (Mori et al., 2008; Bilyachenko et al., 2016; Yalymov et al., 2017; Zhang et al., 2017). Recently, we indicated that Fe(ClO_4_)_2_ salt using H_2_O_2(aq)_ in CH_3_CN can conduct selective oxidation of toluene either for *sp*^2^ C–H bond oxidation to cresols or *sp*^3^ C–H bond oxidation to benzyl alcohol/benzaldehyde (Ramu et al., 2017). Interestingly, the slow addition of H_2_O_2(aq)_ to the reaction mixtures led to π-activation (~70%) for cresols/methyl-*p*-benzoquinone (BQ) formation, whereas the fast addition of H_2_O_2(aq)_ resulted in the major production *sp*^3^ C–H bond oxidation at the methyl substituent (Ramu et al., 2017). Further scrutinizing the reaction mixture by the electron microscopic (EM) study revealed small nanoparticle formation with the sizes of 2–10 nm in the first 2 h with the slow addition of H_2_O_2(aq)_ (Supplementary Figure 1A, Supplementary Information) and relatively larger particles of >1 μm were observed within 2 h after the fast addition of H_2_O_2(aq)_ (Supplementary Figure 1B, Supplementary Information). These particles were evidenced to be consisted of iron oxides from transmission electron microscopy (TEM), energy-dispersive X-ray (Supplementary Figure 2, Supplementary Information), and elemental analysis (Supplementary Table 1, Supplementary Information) study. It is not surprising that with the fast addition of H_2_O_2(aq)_, exothermic heat accumulated for the micro-size iron oxide nanoparticle formation, substantial amounts of HO·/HOO· were generated and the overall *sp*^3^ oxidation products can achieve >95%. To further examine the production of benzyl hydroperoxide, the treatment of PPh_3_ can further assist for its quantification (Shul'pin et al., 2010). Once the reaction is harvested, a very small amount of benzyl hydroperoxide species was observed in rapid addition of H_2_O_2(aq)_. However, with slow addition of H_2_O_2(aq)_, ~45% *sp*^3^ C–H bond oxidation products were observed, and the ratios for benzyl hydroperoxide, benzaldehyde, and benzyl alcohol were identified to be 18, 25, and 2%, respectively (Supplementary Table 2, Supplementary Information). Nevertheless, the selectivity for *sp*^2^ carbon oxidation to cresols/methyl-*p*-BQ formation, with and without treatment of PPh_3_ after the reactions, did not vary much with rapid or slow additions of H_2_O_2(aq)_.

The study using [4-^2^H_0, 1_]toluene for its oxidation catalyzed by Fe(ClO_4_)_2_ catalyst using H_2_O_2_ in CH_3_CN exhibited high NIH-shift ratios of 83–86% (Ramu et al., 2017). On further examination using the resulted iron nanoparticles, the selectivity for *sp*^2^ C–H bond oxidation in cresols/*p*-BQ formations can still reach 65% (Supplementary Table 3, Supplementary Information) where the obtained NIH-shift ratio of deuterium remaining for *p*-cresol product is 81% (Supplementary Information). The outcome here implicated that the activation of aromatic π -bonds for arene oxide or σ-complex may be mediated by iron-based oxygenated species (Kudrik and Sorokin, 2008; Thibon et al., 2012; Raba et al., 2014) that was presumably arisen from the resultant particle surface of the iron nanoparticle.

## Highly Selective Oxidation of Benzene to *p*-Benzoquinone (*p*-BQ) by Copper oxide Nano-catalyst

Multi-copper complexes including cage compounds were employed for selective oxidation of alkanes and aromatics with H_2_O_2(aq)_ in CH_3_CN (You et al., 2014; Kulakova et al., 2017, 2019). Shul'pin and co-workers further reported a double oxidation from benzene to *p*-BQ with molar ratio of ~1:1 (PhOH:*p*-BQ) using Cu(ClO_4_)_2_ (Shul'pina et al., 2008). Similarly, a series of tri-copper cluster complexes achieved an efficient conversion of benzene to *p*-hydroquinone with percentage selectivity of 60–98% (Nagababu et al., 2012). Recently, we have discovered and studied copper oxide–based nano-catalysts for the efficient and selective double oxidation from benzene to *p*-BQ. During the process, the addition of optimized amount of H_2_O in the CH_3_CN solution with copper nanoparticles yielded higher overall catalytic efficiency yield based on consumed H_2_O_2_ value (98%), and better selectivity for *p*-BQ (77%), than other volumes of water (Wanna et al., 2019b). Most importantly, we were able to recycle the catalyst at least three times without significantly losing activity and *p*-BQ selectivity.

**Figure 3:**
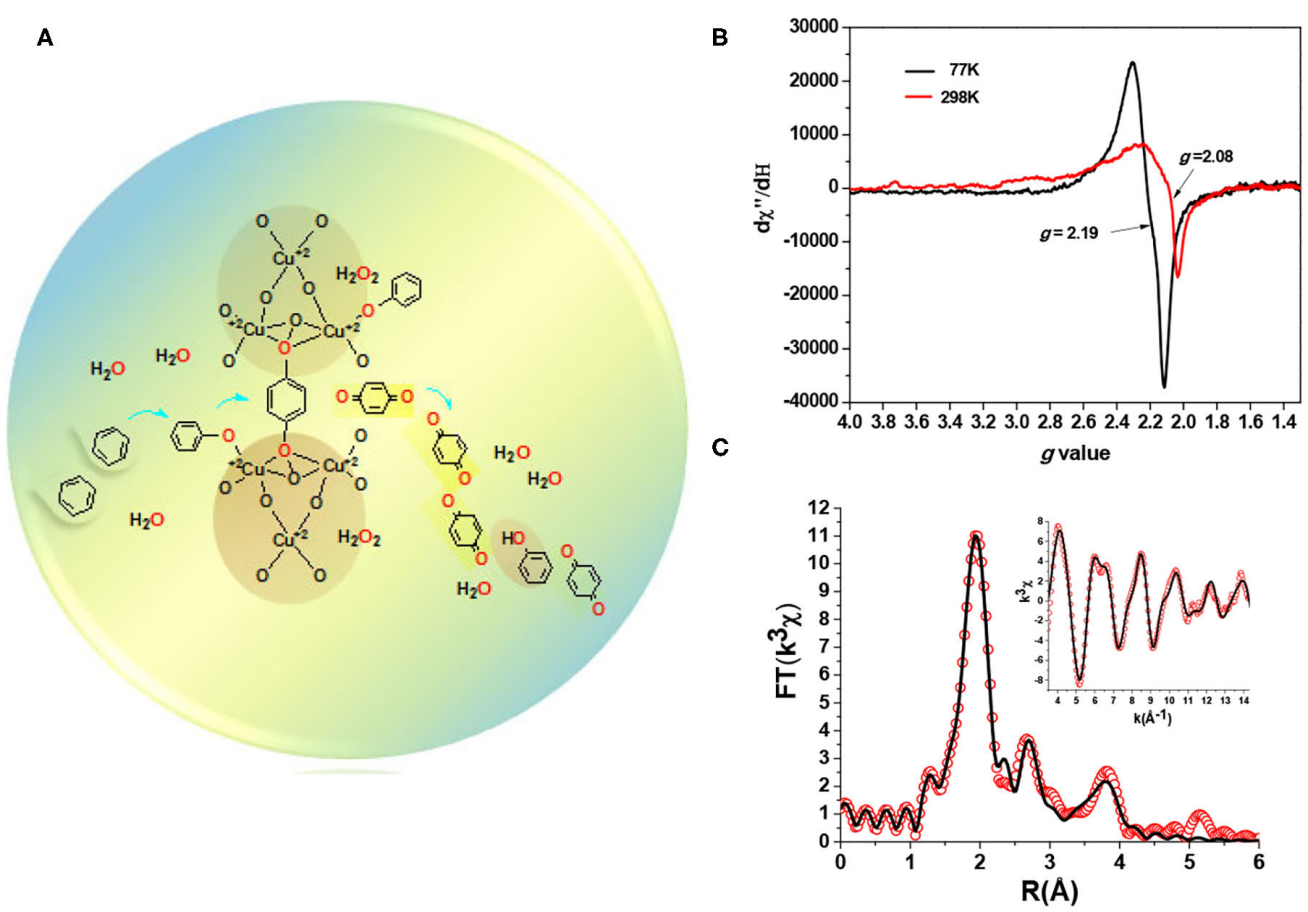
**(A)** The proposed reaction sphere in recyclable copper nano-catalyst with the active site for selective oxidation of benzene to *p*-benzoquinone (*p*-BQ) using H_2_O_2(aq)_ in CH_3_CN; **(B)** EPR spectra of copper oxide nanoparticles in KBr measured at 77 and 298 K, respectively; **(C)** The Cu EXAFS (inset, red circle) and their Fourier transforms (red circle) as well as the corresponding best fits (black solid lines) of copper oxide nanoparticles using trinuclear copper cluster as the structural model (Chan et al., 2013). Reproduced with permission from Wanna et al. (2019b). Copyright 2019 Elsevier B.V.

The EPR spectrum of copper oxide nanoparticles at 298 K indicated an isotropic signal, reminiscent of a previous study of pMMO with trinuclear copper cluster feature (Nguyen et al., 1994), which was significantly broadened and presented relatively poor signal-to-noise ratio than the one at 77 K (Figure 3B) (Wanna et al., 2019b). The result here reveals substantial dynamic motions around the metal centers, which are behaving like metal active sites in the metallo-monooxygenases (Chen et al., 2014; Wang et al., 2017; Yang et al., 2017). In addition, the corresponding EXAFS data fittings (*k*^3^-weighted) using two structural models of copper oxide and trinuclear copper cluster model (Cu_2.8_O_4.6_), respectively, can both give reasonable fits of *R*_fit_ = 0.027 and 0.15%, respectively (Wanna et al., 2019b), which indicated that a trinuclear copper cluster feature is manifested from the obtained oxide materials (Figure 3). Especially, the specific Cu–Cu scattering in a distance of ~3.7 Å would presumably result in Cu^2+^(μ-(η^2^:η^2^)-peroxo)Cu^2+^ (Cu–Cu distance ~3.6 Å) that can potentially reach an equilibrium of a Cu^3+^(μ-O)_2_Cu^3+^ structure (Lewis and Tolman, 2004; Mirica et al., 2004; Ottenwaelder et al., 2006). The formed possible intermediate of Cu^2+^Cu^2+^(μ-O)_2_Cu^3+^ can exhibit much lower activation energy for a facile oxo-transfer toward the inert C–H bond (Chen and Chan, 2006). It is not surprising that a benzene molecule can therefore easily possess an arene oxide intermediate *via* OAT followed by subsequent NIH rearrangement to obtain PhOH from benzene. In fact, the recycled copper nanoparticles performed with reasonable efficiency in toluene oxidation. The NIH-shift ratios obtained from the deuterated *p*-cresol products was 54%, which displayed that the solid-state surface of copper nanoparticles can in general oxidize *ortho-* or *para*- (*sp*^2^) C–H bonds of toluene *via* the formation of arene oxide intermediate followed by an NIH rearrangement process.

## The Mixed Valence Sites of V^4+^/V^5+^ Stabilized by PCA in the V_2_O_5_ for Selective Oxidation of Benzene to Phenol With High Efficiency

**Figure 4:**
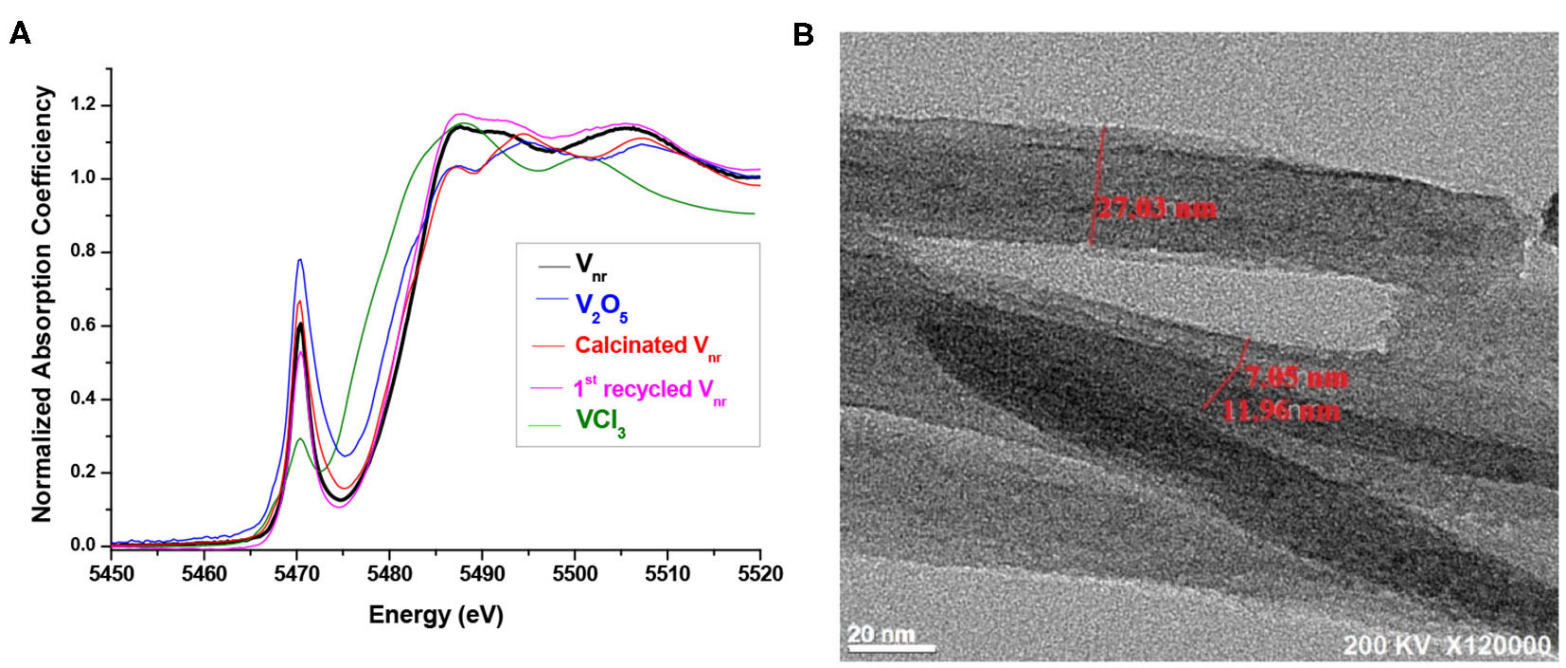
**(A)** Normalized V K-edge X-ray absorption near edge structures (XANES) of vanadium oxide nanorod (**V**_**nr**_) (*black line*) and its calcined materials (**V**_**nr**__(cal)_, *red line*), V_2_O_5_ (*blue line*), VCl_3(aq)_ (*green line*), and the 1^st^ recycled **V**_**nr**_ (*magenta line*) recycled from the **V**_**nr**_ catalyst after the catalytic reaction of benzene. **(B)** The high-resolution TEM image of rod-shaped **V**_**nr**_. Reproduced with permission from Wanna et al. (2019a). Copyright 2019 The Royal Society of Chemistry.

Vanadium-based polyoxometalates have been extensively used as green heterogeneous catalysts with high catalytic activity for selective oxidation of styrene to benzaldehyde (Zhou et al., 2020), benzene to PhOH (Li et al., 2020), and mono-substituted benzene to *p*-PhOH (Kamata et al., 2012). Kirillov and Shul'pin (2013) established a variety of vanadium complexes to catalyze aerobic and/or H_2_O_2_ hydroxylation of benzene to phenol and toluene to a mixture of cresols in CH_3_CN. Mandatory promoters such as pyridine, pyrazine-2-carboxylic acid (PCA), and acetic acid were used as co-catalysts to prompt the oxidation reactions. We recently reported a unique vanadium nanorod (**V**_**nr**_) catalyst (Figure 4B) for selective oxidation of benzene to PhOH (>87%) with minor *p*-BQ formation in CH_3_CN. The presence of PCA, interacting V^+4^/V^+5^ in **V**_**nr**_, can serve as Brønsted acid site to improve activity for PhOH production (Liu et al., 2017; Zhao et al., 2018). In fact, PCA ligand can serve as a stabilizer of the transition state involving V^4+^ species for the H-transfer (Kirillova et al., 2009) and the reduction of V^5+^ to V^4+^ by PCA with H_2_O_2(aq)_ assists in accumulating more redox active centers (Shul'pin et al., 1999). During the detailed characterization of V_2_O_5_-related materials, including the **V**_**nr**_ catalyst prepared from VCl_3_, we found that lower pre-edge peak intensity of XAS data at ~5,470 eV together with XPS data analysis indicated **V**_**nr**_ catalyst exhibited higher V^+4^/V^+5^ ratio than the “calcined **V**_**nr**_” (**V**_**nr**__(cal)_) and commercial V_2_O_5_ (Figure 4A). For the efficient conversion of benzene to PhOH, assisted by PCA, the vanadium materials exhibit high dependence on V^+4^/V^+5^ ratios (Table 1).

**Table 1:** The summary of X-ray photoelectron spectroscopy (XPS) and X-ray absorption spectroscopy (XAS) data of vanadium oxide catalysts including **V**_**nr**_ and its reactivity parameters.

**Catalysts**	**XPS**	**Normalized XAS intensity**	**Gain factor^**a**^**	**Selectivity^**b**^ (%)**	**TONs^**c**^ of PhOH**
	**V^**4+**^/V^**5+**^ ratio**	**5,470 eV**			
**V**_**nr**_	0.301	0.61	0.13	87	11.7
**V**_**nr**_(PCA)	0.301	0.61	0.64	95	25.5
1^st^ recycled **V**_**nr**_ (PCA)	0.639	0.53	0.31	91	20.3
**V**_**nr**__(cal)_ (PCA)	0.218	0.67	0.23	94	17.1
V_2_O_5_ commercial (PCA)	0.118	0.78	0.09	95	9.6

a *Gain factor = (mol of oxygenated product)/(mol of H_2_O_2_ consumed). ^b^Selectivity of PhOH = [(PhOH) (mol)/[p-BQ + PhOH] (mol)] × 100. ^c^Turnover number (TON) for PhOH was determined by calculating the number of millimoles of product per millimole of catalyst (based on V content)*.

**V**_**nr**_ catalyst can also conduct toluene oxidation and its selective *sp*^2^ C–H bond oxidation or π-activation for cresols/methyl-*p*-BQ formation up to 52% assisted by PCA (Wanna et al., 2019a). For 48% *sp*^3^ C–H bond oxidation products, in comparison with the product ratio by the addition of PPh_3_ after the reaction, the ratios for benzaldehyde and benzyl hydroperoxide were identified to be 23 and 25%, respectively (Supplementary Table 2, Supplementary Information). In addition, high NIH rearrangement ratio of 80% suggests that the reaction mechanism for *sp*^2^ C–H bond oxidation/π activation of toluene, presumably, also contributed partly to the catalytic oxidation of benzene to phenol, mainly *via* an electrophilic OAT of vanadium hydroperoxide or high valent vanadium oxo intermediates. The oxidation of benzene to PhOH catalyzed by **V**_**nr**_ catalyst using ${\text{H}}_{2}^{18}$O_2(aq)_ as an oxidant displayed 68% ^18^O-enriched PhOH; however, there was negligible enrichment using ${\text{H}}_{2}^{16}$O_2_ and ^18^O_2_. The results implicate H_2_O_2(aq)_ as the essential oxidant for OAT mediated by the vanadium oxygenated species.

## Characterizations and Common Features of the Fe/Cu/V Nano-Catalysts

The Fe, Cu, and V oxide nano-catalysts as a type of metal oxide material possessed metal-oxygen/hydroxide components that can be polymerized and form active hybrid materials with oxygenated hydrocarbons (Supplementary Figures 1–7, Supplementary Information). N_2_ adsorption–desorption isotherms and XRD studies reveal the porous structure and crystalline feature of these catalysts that formed from the core metal oxide with the organic linker as a framework. These three catalysts similarly possessed mesoporous structure in a pore size range of 1.5–30 nm and BET surface area of each was in the range of 20–30 m^2^/g that can be essential for catalytic activation of the small aromatic compounds. The mesopore structure of the catalysts has a type of slit-like pore formed by the effect of substrate (benzene/toluene) acting as template while preparing *in situ* (Rouquerol et al., 1994; Thommes et al., 2015; Wanna et al., 2019a,b). The porous property observed in these Fe/Cu/V nano-catalysts contributed to the improvement of benzene oxidation efficiency using H_2_O_2(aq)_ with additional H_2_O proportions in CH_3_CN (Ramu et al., 2017; Wanna et al., 2019b), which is consistent with the effect of water-assisted benzene oxidation catalyzed by the molecular iron complex encapsulated in the zeolite (Yamaguchi et al., 2015). In addition, the powder XRD and selected area (electron) diffraction SAD-TEM analysis for **V**_**nr**_ catalyst indicated that the crystal grew longer to rod shape with preferential (220) orientation that can be crucial for the related additional activities (Berenguer et al., 2017). The crystalline feature of Fe and Cu catalysts also exhibited a distinctive crystal arrangement that directly corresponded to the nature of the parental metal-oxide materials.

Thermogravimetric analysis study of the organic composition of the nano-catalysts indicates that it accounts for 10–50% and plays key roles for structural framework formation and selective catalytic activity (Supplementary Figures 3, 5, 7, Supplementary Information) (Zoubi et al., 2009; Wanna et al., 2019a,b). Calcination of these catalysts at 500°C under N_2_ completely decomposes the organic linker, collapses the structural framework, and changes the surface morphology that causes a decline in catalytic activity significantly. In the XPS investigation of the catalysts' surface, beside the common metal-oxide/hydroxide functional groups, we identified a hydrocarbon group that offers hydrophobic surfaces (Wanna et al., 2019a,b) that are crucial for high dispersion of the nanoparticles and surface–substrate interaction (Wu et al., 2006; Biesinger et al., 2007; Dong et al., 2016). The central metal-oxide cluster in support with the organic linker forms the surface atomic structure that can be active for catalytic application. In addition to this, XPS and XAS studies show coexistence of the mixed valance states such as Fe^2+^/Fe^3+^, Cu^+^/Cu^2+^, and V^4+^/V^5+^ that are essential for C–H or π-bond activation.

## Discussion

Overall, the study provides an efficient strategy that accumulates active Fe, Cu, and V oxide species of organic–inorganic hybrid nano-catalysts, respectively, through the addition of 35% H_2_O_2(aq)_ to the Fe/Cu perchlorate and VCl_3_ in CH_3_CN for the selective oxidation of simple aromatics (Ramu et al., 2017; Wanna et al., 2019a,b). Interestingly, these metal nanoparticles can be further recycled several times that can carry out the selective catalytic oxidation of benzene to PhOH or *p*-BQ and toluene to cresols/methyl-*p*-BQ (*sp*^2^ C–H bond oxidation) or benzyl alcohol/benzaldehyde (*sp*^3^ C–H bond oxidation). This indicates that, in the presence of organic residues including CH_3_CN, benzene, and/or toluene, the oxidant of H_2_O_2(aq)_ can not only accumulate HO·/HOO· but is also essential for the self-assembly of the heterogeneous metal-oxide hybrid catalyst formation with active metal oxygenated species.

CH_3_CN is a polar solvent that can be miscible with polar H_2_O_2_ and H_2_O as well as the non-polar aromatic substrates, leading to oxygenate formation. When the metal salts dissolved in the reaction mixtures of aromatics, the free Cu/Fe/V ions presumably coordinate with CH_3_CN serving as a ligand to form metal complexes (Rach and Kühn, 2009; Kulakova et al., 2017). As **V**_**nr**_ catalyst can be directly prepared from H_2_O_2(aq)_ in CH_3_CN without any additional substrates (Wanna et al., 2019a), we therefore surmise that these CH_3_CN-based metal complexes in the presence of H_2_O_2(aq)_ can occur and are essential to assist the metal oxide polymerization/formation.

In addition, it has already been known that catalytic aromatic hydroxylation with H_2_O_2(aq)_ is the cause of further oxidation of initially formed phenols and the appearance of tar (Olah et al., 1981; Kholdeeva, 2015). As long as we take into consideration this additional factor in a heterogeneous platform for the metal catalyzed oxidation or the reaction condition for Fenton's chemistry, the accumulated ROS exerted on the oxide surface may not be considered as diffusible radicals anymore. The Cu and Fe nanoparticles can achieve the oxidation of toluene for *o,p*-cresols in 75% selectivity whereas for the **V**_**nr**_ materials, it is ~52%. The NIH-shift ratios obtained from the mechanistic probe of [4-^2^H_0, 1_]toluene catalyzed by Fe, Cu, and V nano-catalysts are ~85, 57, and 80%, respectively (Ramu et al., 2017; Wanna et al., 2019a,b). The results further support that the oxidation using H_2_O_2(aq)_ in CH_3_CN catalyzed by the metal oxide nano-catalysts can give rise to reactivity from the specific Fe^IV^ = O species on poly-iron oxo clusters (Enami et al., 2014) as evidenced from EM studies, tri-copper cluster moiety that existed in the copper oxide assembled nanoparticles (Wanna et al., 2019b), and crucial V^4+^/V^5+^ redox sites stabilized by PCA (Wanna et al., 2019a), which can significantly undergo OAT process for aromatics activation *via* the arene oxide intermediates (Guroff et al., 1967; Jerina and Daly, 1974; Bassan et al., 2003).

In summary, highly dispersed active Fe, Cu, and V nano-catalysts with H_2_O_2_/O_2_, in a heterogeneous platform, can drive selective oxidation of simple aromatics such as benzene and toluene. Furthermore, these unique catalysts effectively tuned *sp*^2^ *vs. sp*^3^ C–H bond hydroxylation of toluene and achieved selective double oxidation of benzene to *p*-BQ. In many cases, the reaction mechanism proceeds significantly through OAT process *via* the formation of the arene oxide intermediate, although the free radicals that initiated oxidation of C–H bond also participate. These nano-catalysts prepared *in situ* and composed of organic–inorganic hybrid exhibited recyclable properties, controlled selectivity, higher activity, and were greener than their bulk oxide counterparts, which make them potentially promising for large-scale application.

## Author Contributions

WW, DJ, NT, and RR conducted the research of catalytic oxidative conversion of simple aromatics. WW, DJ, NT, RR, and Y-FT carried out the identification and characterization of the metal oxide materials. SY organized the research. WW, DJ, NT, and SY wrote the paper. All authors contributed to the article and approved the submitted version.

## Conflict of Interest

The authors declare that the research was conducted in the absence of any commercial or financial relationships that could be construed as a potential conflict of interest.
