# Supplementary material for: Selective Oxidation of Simple Aromatics Catalyzed by Nano-Biomimetic Metal Oxide Catalysts: A Mini Review
Source: Front Chem. 2020 Oct 26;8:589178. doi: 10.3389/fchem.2020.589178 (PMC7649321; doi:10.3389/fchem.2020.589178)
Supplement: Supplementary file 1 [file Presentation_1.pdf]

Supplementary Information

Selective Oxidation of Simple Aromatics Catalyzed by Nano-Biomimetic Metal Oxide Catalysts: A Mini Review†

Wondemagegn H. Wanna[1‡], Damodar Janmanchi[1‡], Natarajan Thiyagarajan[1‡], Ravirala Ramu[1,2], Yi-Fang Tsai[1] and Steve S.-F. Yu[1*]

A. Tables

Table 1. The Elemental Analysis (EA) data of the Fe, Cu and V-oxide nano-catalyst.

	Oxide nano-catalysts EA (Weight %)		
Elements	Cu	Fe	V
Metal	27.3±1.3	26.44±3.2	71.44±2.7
O	39.9	40.6	22.60
C	16.7	21.5	3.50
N	2.2	1.5	0.89
H	1.5	2.9	1.57
Total	100	100	100

Table 2. Oxidation of toluene to benzaldehyde, benzyl hydro-peroxide, benzyl alcohol, methyl-*p*-benzoquinone, *o*-cresol, and *p*-cresol were estimated according to PPh_3 treatment.[1-4]

Catalyst	**Benz-aldehyde** (%)	**Benzyl Hydro-peroxide**(%)	**Benzyl Alcohol** (%)	**Methyl-*p*-Benzo-quinone** (%)	***o*-cresol** (%)	***p*-cresol** (%)	sp^2/sp^3 [%][b]
$Fe(ClO_4)_2 \cdot xH_2O$ Slow addition H_2O_2	**25**	**18**	**2**	**7**	**24**	**24**	**55**
$Fe(ClO_4)_2 \cdot xH_2O$ Rapid addition H_2O_2	**82**	**3**	**11**	**1**	**2**	**1**	**4**
Copper Nanoparticle[a]	**11**	**N.D.**	**2**	**32**	**25**	**20**	**77**
$\mathbf{V_{nr}}$	**23**	**25**	**0**	**10**	**22**	**20**	**52**

[a]The product ratios of toluene oxidation were not calibrated by the treatment of PPh_3; [b]Selectivity for sp^2 = [ring-oxygenated products (mol) / all oxidation products (mol)] × 100; N.D.: not detected.

Table 3. Fe-catalyzed oxidation of toluene to benzyl alcohol (**a**), benzaldehyde (**b**), *o*-cresol (**c**), *p*-cresol (**d**), and methyl-*p*-benzoquinone (**e**) using recycled Fe nanoparticles (0.05 mol%) *via* the slow addition of $H_2O_{2(aq)}$.[a]

Entry	Catalyst	**a:b:c:d:e** [TON][b]					Total TON[c]	Y_S [%][d]	Y_O [%][e]	Selectivity for *sp*^{2} [%][f]
1	Recycled Fe Nanoparticle	7.3	32.3	38.7	30.1	4.4	113	5.8	4.4	65

[a]Conditions: Total volume of the reaction solution was 3 mL (Addition of 100 μL trifluoroacetic acid (TFA) is essential for suspension of the recycled nanoparticles); Solvent: acetonitrile; Catalyst: 0.05 mol% Fe; Substrate: toluene (3.87 mmol); Oxidant: 35% aq. H_2O_2 (4.64 mmol; slow addition); Reaction time: 15 h; Temperature: 25°C; Determined by GC and GC/MS analysis using nitrobenzene as an internal standard. [b]Turnover number (TON) = [product (mol) / catalyst (mol)]; [c]The total TON of products (a+b+c+d+e); [d]Y_S [%] = [products (mol) /initial substrate (mol)] x 100; [e]Y_O [%] = [products (mol) / initial H_2O_2 (mol)] × 100.; [f]Selectivity for *sp*^{2} = [ring-oxygenated products (mol) / all oxidation products (mol)] × 100.

B. Determination of NIH-shift ratios of [4-$^2H_{0,1}$]toluene catalyzed by Fe recycled nanoparticles in CH_3CN by the addition of $H_2O_{2(aq)}$ using GC-MS

1 mg iron nanoparticle (0.05% mol) in H_2O_2-H_2O-CH_3CN at room temperature.

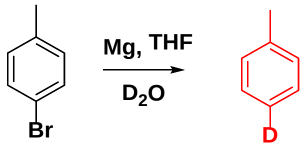

MW = 93 (73% deuterium enrichment)
MW: Molecular Weight

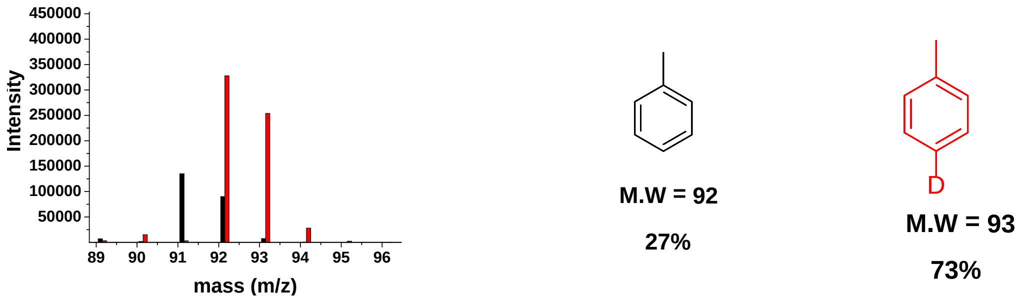

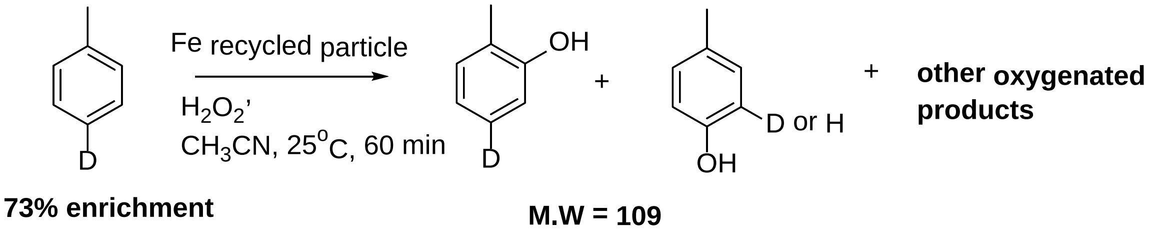

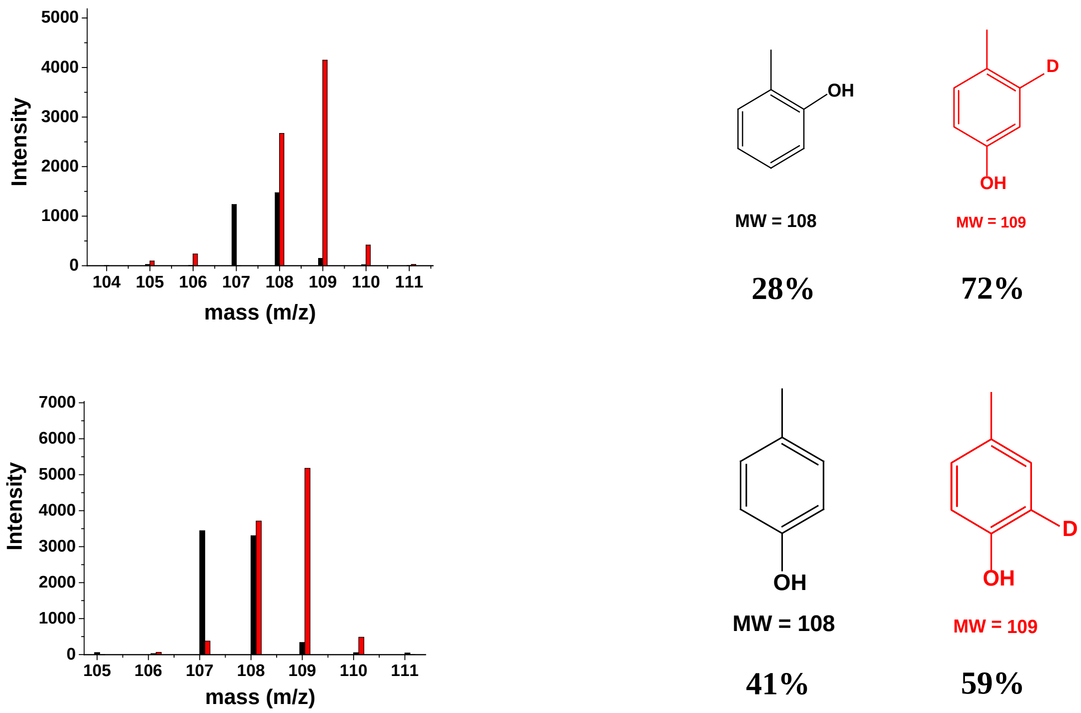

NIH rearrangement ratio = 59%/73% = 81%

C. TEM and SEM/EDX study of the Fe oxide, Cu oxide and V oxide accumulated from the reaction mixtures in CH_3CN by the addition of $H_2O_{2(aq)}$.

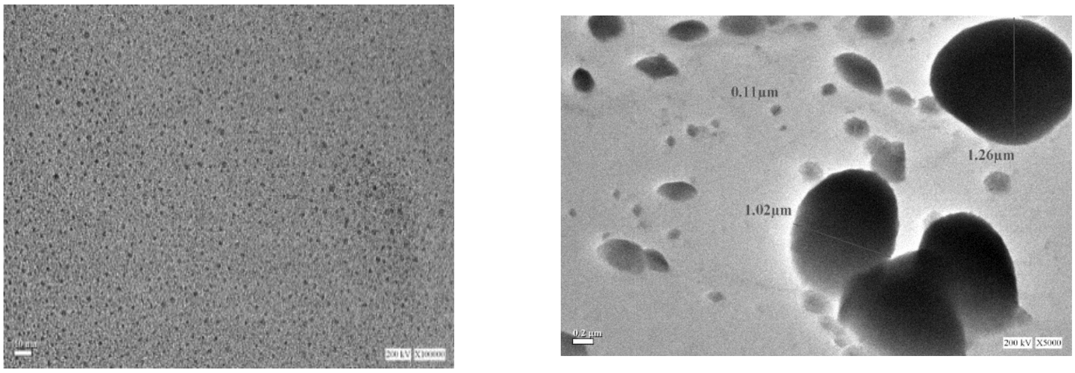

A **B**

Figure 1. TEM studies of the formation of Fe particles by (a) a slow addition of 35% $H_2O_{2(aq)}$ (5.16 mmol) for 1 h to a reaction mixtures of toluene (3.87 mmol) in CH_3CN (total in 3 mL); (b) a rapid addition of 35% $H_2O_{2(aq)}$ (5.16 mmol) within 30 sec to toluene (3.87 mmol) in CH_3CN (3 mL). The samples were collected after the reactions were proceeded for 2 h at room temperature.

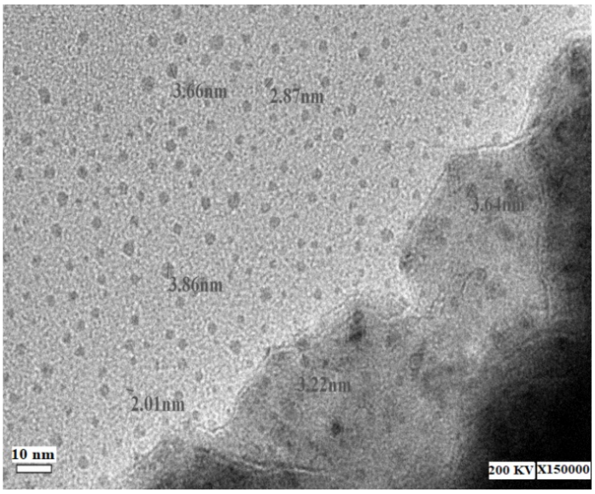

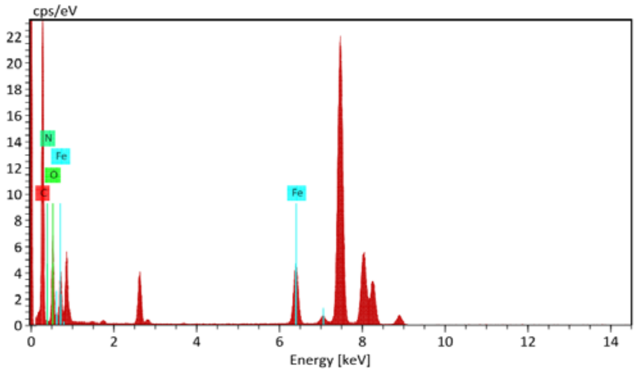

Element	At. No.	Netto	Mass Norm. [%]	Atom [%]	rel. error [%] (1 sigma)
Carbon	6	79972	71.53	87.09	3.06
Nitrogen	7	533	0.36	0.37	12.31
Oxygen	8	23912	7.94	7.26	3.38
Iron	26	44511	20.18	5.28	3.17
		Sum	100.00	100.00	

Figure 2. Transmission electron microscopy (TEM) image and energy dispersive X-ray (TEM-EDX) data of Fe oxide nanocatalyst.

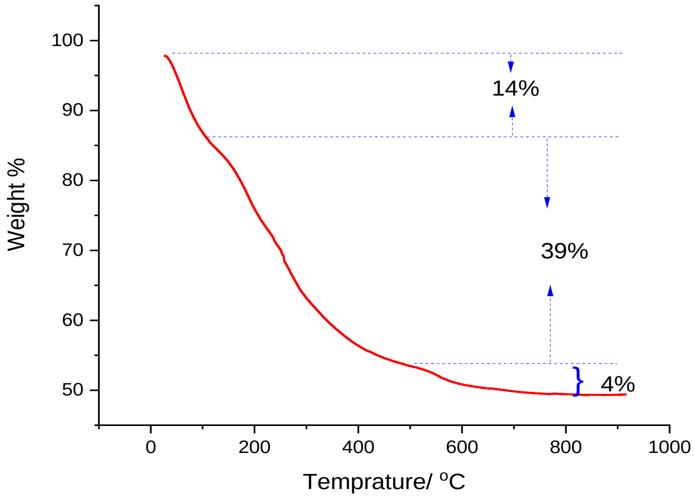

Figure 3. Thermo-gravimetric analysis (TGA) diagram for iron oxide nano-catalyst, the weight loss was measured in a graph with two inflection points at 500 °C (36.5%) and 550 °C (4.5%), respectively.

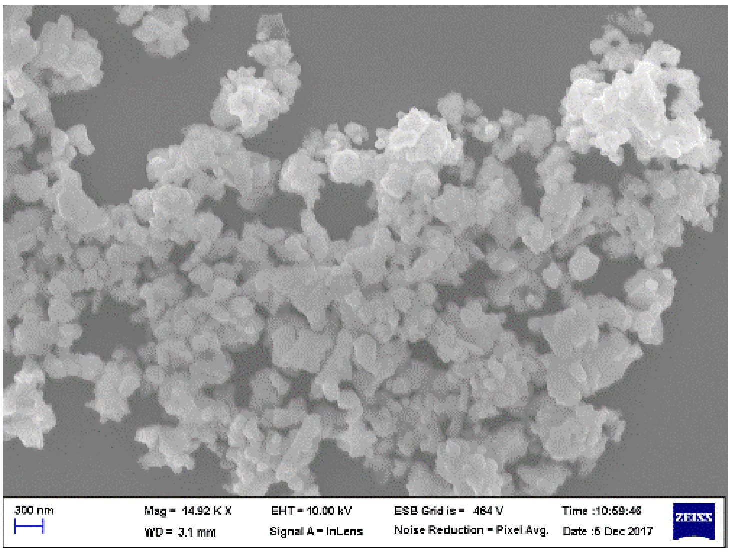

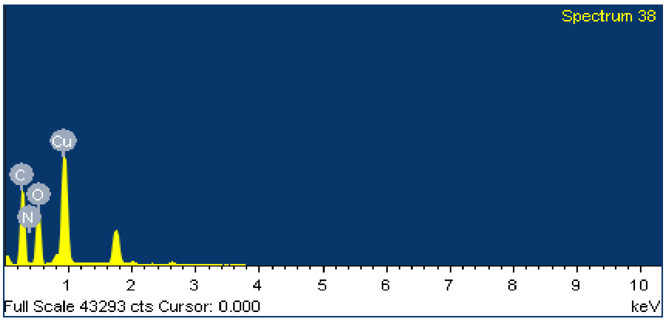

Elements	Weight %	Atomic %
C K	34.28	58.04
N K	1.57	2.29
O K	20.14	25.60
Cu L	44.00	14.08
Total	100	

Figure 4. Scanning Electron Microscopy (SEM) image and energy dispersive X-ray (SEM-EDX) data of Cu oxide nanocatalyst.

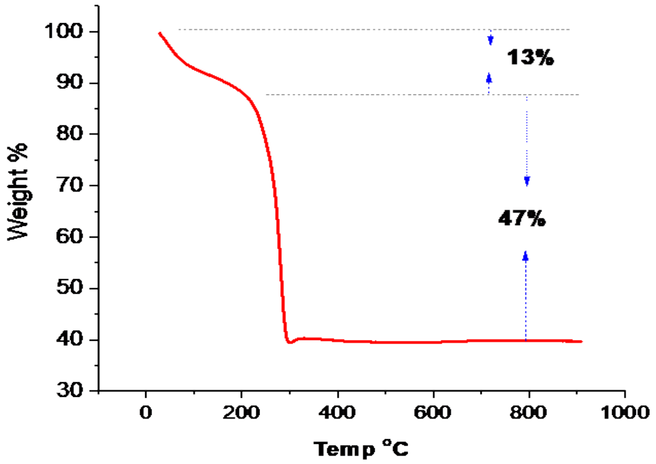

Figure 5. TGA diagram of Cu-oxide nano-catalyst prepared *in situ* from benzene oxidation reaction in $H_2O_{2(aq)}$-CH_3CN system.

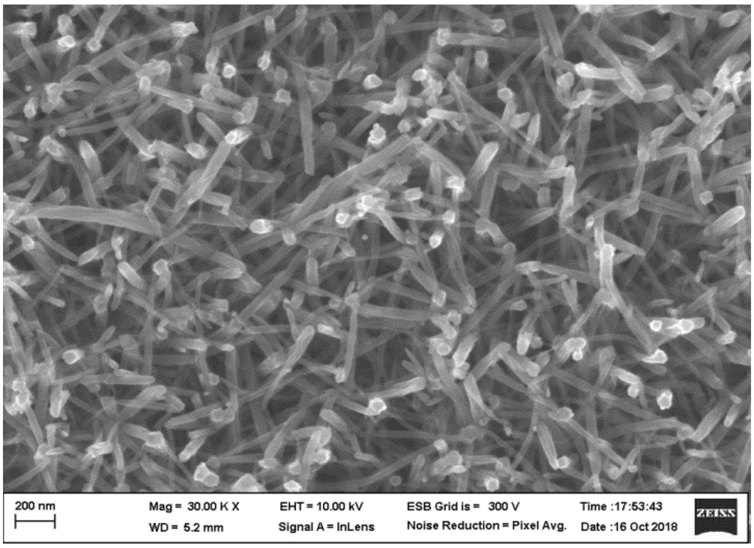

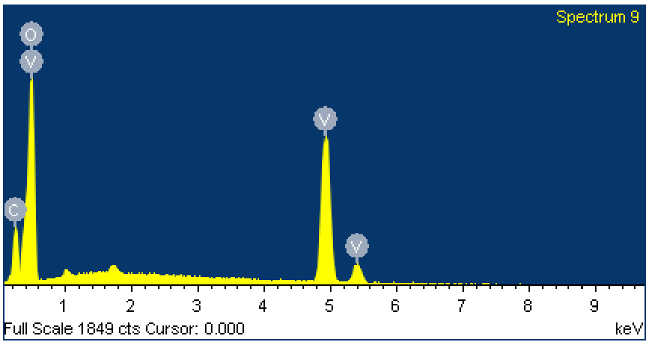

Element	Weight %	Atomic %
C K	4.98	11.83
O K	28.50	50.87
V K	66.52	37.29
Totals	100.00	

Figure 6. SEM-EDX data of Vanadium oxide nanorod (**V**$_{nr}$) nanocatalyst.

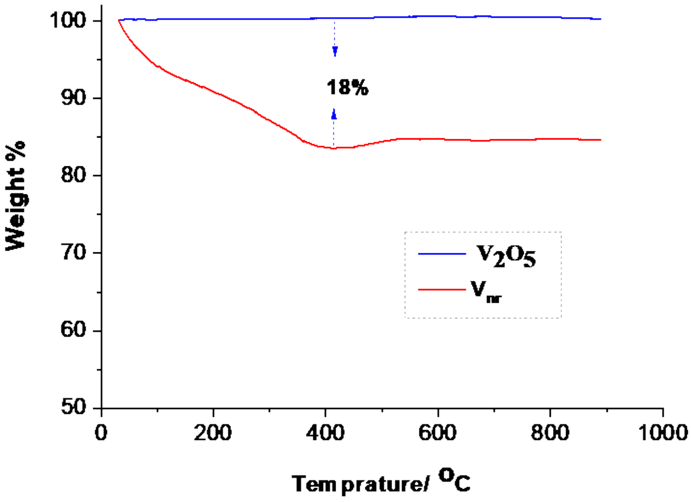

Figure 7. TGA diagram of vanadium oxide nanorod (**V**$_{nr}$) catalyst and commercialized bulk V_2O_5 powder.

Experimental

The iron oxide nano-catalyst prepared from the $Fe(ClO_4)_2$ precursor *in situ* reaction of the aromatic oxidation in $H_2O_{2(aq)}$ -CH_3CN (**Figure 1**). The selective toluene oxidation, catalyzed by 10 mg $Fe(ClO_4)_2$ (2 mol%), with the slow addition of $H_2O_{2(aq)}$ in CH_3CN (total in 3 mL), will lead to sp^2 C–H bond oxidation or π-activation (~70%) for cresols/2-methyl-*p*-benzoquinone (*p*-BQ) formation. The mechanistic study used the substrate of [4-$^2H_{0,1}$]toluene for its oxidation catalyzed by both the slow addition of $H_2O_{2(aq)}$ and 2 mol% of $Fe(ClO_4)_2$, and fast addition of $H_2O_{2(aq)}$ and 0.1 mol% of $Fe(ClO_4)_2$ in CH_3CN.

The process of benzene oxidation catalyzed by $Cu(CH_3CN)_4ClO_4$ using $H_2O_{2(aq)}$ as an oxidant, besides the selectivity for *p*-BQ formation, we have also observed copper oxide nanoparticles generation, detected by using Transmission Electron Microscopy (TEM) (~4–6 nm).[3] These nanoparticles can be collected, recycled for several times and render better catalytic activity. The addition of various amounts of H_2O (0–500 μL) to the recycled copper oxide catalyst for the benzene oxidation to *p*-BQ in the 35% $H_2O_{2(aq)}$-CH_3CN (3 mL in total) for 24 h was examined. The study of copper oxide nano-catalyst chemical and structural compositions were reported. In a similar strategy, a vanadium oxide nanorod (**V_{nr}**) catalyst, prepared through facile crystallization method from the VCl_3 reaction mixtures in CH_3CN by the addition of $H_2O_{2(aq)}$. The resulted **V_{nr}** materials performed selective oxidation of benzene to phenol in CH_3CN at 25 ^oC.[4]

References

1. Shul'pin, G.B., Kozlov, Y.N., Shul'pina, L.S., and Petrovskiy, P.V. (2010). Oxidation of alkanes and alcohols with hydrogen peroxide catalyzed by complex $Os_3(CO)_{10}(\mu\text{-}H)_2$. *Appl. Organometal. Chem.* 24**,** 464-472.

2. Ramu, R., Wanna, W.H., Janmanchi, D., Tsai, Y.-F., Liu, C.-C., Mou, C.-Y., and Yu, S.S.-F. (2017). Mechanistic study for the selective oxidation of benzene and toluene catalyzed by $Fe(ClO_4)_2$ in an H_2O_2-H_2O-CH_3CN system. *Mol. Catal.* 441**,** 114-121.
3. Wanna, W.H., Ramu, R., Janmanchi, D., Tsai, Y.-F., Thiyagarajan, N., and Yu, S.S.F. (2019). An efficient and recyclable copper nano-catalyst for the selective oxidation of benzene to p-benzoquinone (*p*-BQ) using $H_2O_{2(aq)}$ in CH_3CN. *J. Catal.* 370**,** 332-346.
4. Wanna, W.H., Janmanchi, D., Thiyagarajan, N., Ramu, R., Tsai, Y.-F., Pao, C.-W., and Yu, S.S.-F. (2019). Selective catalytic oxidation of benzene to phenol by a vanadium oxide nanorod (Vnr) catalyst in CH_3CN using $H_2O_{2(aq)}$) and pyrazine-2-carboxylic acid (PCA). *New J. Chem.* 43**,** 17819-17830.
